# Idiopathic Thrombocytopenic Purpura Misdiagnosed as Hereditary Angioedema

**DOI:** 10.1155/2015/934247

**Published:** 2015-12-24

**Authors:** Michelle Fog Andersen, Anette Bygum

**Affiliations:** ^1^Department of Otorhinolaryngology, Head and Neck Surgery, Køge Hospital, Lykkebaekvej 1, 4600 Køge, Denmark; ^2^HAE Centre Denmark, Department of Dermatology and Allergy Centre, Odense University Hospital, Sdr. Boulevard 29, Entrance 142, 5000 Odense C, Denmark

## Abstract

Hereditary angioedema is a rare, but potentially life-threatening genetic disorder that results from an autosomal dominant trait. It is characterized by acute, recurrent attacks of severe local edema, most commonly affecting the skin and mucosa. Swelling in hereditary angioedema patients does however not always have to be caused by angioedema but can relate to other concomitant disorders. In this report we are focusing on misdiagnosis in a patient with known hereditary angioedema, whose bleeding episode caused by idiopathic thrombocytopenic purpura was mistaken for an acute attack of hereditary angioedema. The case illustrates how clinicians can have difficulties in handling patients with rare diseases, especially in the emergency care setting.

## 1. Introduction

Hereditary angioedema (HAE), originally described by Quincke in 1882 [1], is a rare genetic disorder characterized by recurrent episodes of subcutaneous and submucosal swellings in any part of the skin, including the gastrointestinal tract and upper airway [2, 3]. The disease is caused by mutations in the gene encoding* SERPING1* causing deficiency of complement C1 inhibitor (C1-INH), a protein involved in the regulation of the complement, kinin-kallikrein, coagulation, and fibrinolytic systems [4]. The deficiency results in uncontrolled activation and release of bradykinin, which causes increased vascular permeability and dilatation with a resulting edema at the affected site [5, 6]. HAE is inherited in an autosomal dominant pattern and is estimated to affect 1 in 50,000 individuals, with no clear sex or ethnic variation [7, 8].

There are two classical types of HAE having identical clinical presentations. HAE type I represents 85% of patients with C1-INH deficiency and is characterized by a decreased production of circulating C1-INH. Patients with HAE type II, approximately 15% of cases, have normal concentrations of C1-INH but a dysfunctional protein, meaning a low functional level to be measured [4]. More rarely seen is a third type of HAE, primarily discovered in women, with normal fully functional C1-INH levels presenting with the typical clinical features of C1-INH deficiency. This type has been associated with mutations in F12, the gene encoding the plasma protease factor XII (FXII) [9].

For many health care professionals, HAE present an ongoing challenge due to the rarity and complexity of the clinical presentations, which may involve most organ systems. In this report we are focusing on misdiagnosis in a patient with known HAE who had a swelling caused by bleeding attributed to idiopathic thrombocytopenic purpura (ITP).

## 2. Case Presentation

A 74-year-old man had recurrent episodic attacks of abdominal pain and swelling involving the upper airways, extremities, and genitals from the age of three, leading to both unnecessary appendectomy and later tracheotomy because of laryngeal edema. However, he was not diagnosed with hereditary angioedema, before the age of 33 years, when he was admitted to the emergency unit with a severe swelling of his face. Biochemical analyses were consistent with HAE type I and later a family investigation disclosed a splice site mutation in the* SERPING1* gene (c. 1250-1G>A), a mutation also found in his son and some of his grandchildren. Since the diagnosis, he has been treated with Danazol and he is currently followed up at the National HAE Centre once yearly. After reducing Danazol to the minimum effective dose (200 mg o.d.), he has an average of three relatively mild attacks per year. The patient is monitored every six months with liver enzymes, lipid profile, complete blood cell count, urinalysis, and liver spleen ultrasound. His breakthrough attacks are treated with injections of C1-INH concentrate (Berinert).

**Figure 1:**
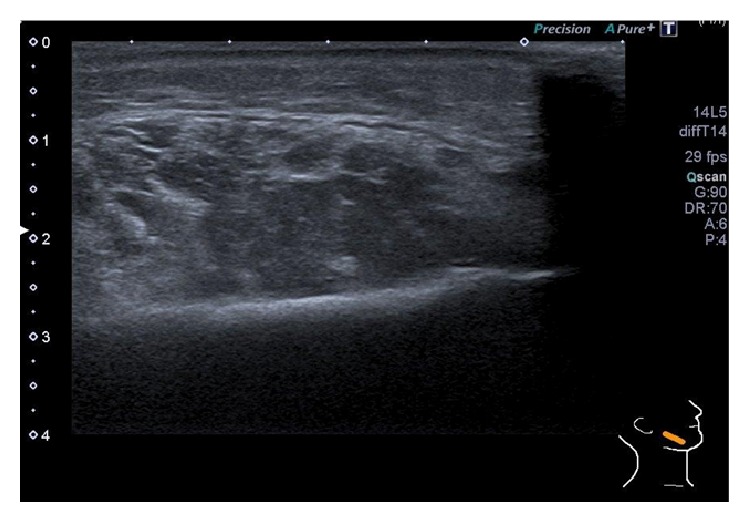
Ultrasound scan of the face shows a subcutaneous haematoma around the right masseter muscle measuring 2 cm in depth. No other pathology was found.

In 2014 the patient presented at the local emergency room (ER) with a severe swelling in the lower part of his face associated with difficulty in swallowing, abdominal pain, and a few red spots on his extremities. He had no stridor or voice changes and there were no signs or symptoms of viral infection before the attack. Apart from the limited purpuric spots, the clinical presentation imitated HAE swelling, which was suggested to be the diagnosis. He was treated with C1-INH concentrate 1000 units intravenously and discharged from the ER. However petechiae evolved over most of his body and suddenly a large haematoma presented spontaneously on his right jaw. He was once again seen at the ER and the clinical examination revealed edema of the right side of his face and lips and inside the mouth and throat. Severe swelling over the right mandibular condyle was found with a palpable, soft, nonfluctuant discolored mass measuring 3 cm in diameter. Multiple small petechiae were detected on the extremities and thorax and in the facial area including mucosal bleeding inside the mouth. Abdominal examination revealed no hepatosplenomegaly or abdominal tenderness and normal bowel sounds were present. Examination of the urine was remarkable for microscopic haematuria. An ultrasound scan was performed showing a subcutaneous haematoma around the right masseter muscle (Figure 1). A computerised tomography (CT) of the neck, thorax, abdomen, and pelvis obtained no pathology besides the haematoma identified on the ultrasound scan. Laboratory tests presented normal white blood cell count and hemoglobin level. His platelet count was significantly low at 3 × 10^9^/L [normal count: 150–450 × 10^9^/L]. Bone marrow aspiration was performed and revealed trilinear marrow hyperplasia with megakaryopoiesis, compatible with idiopathic thrombocytopenic purpura (ITP). It came out that the patient was diagnosed with ITP 10 years earlier, but he had forgot about this former diagnosis and seemingly it was not looked up in the ER. He was referred to a medical department and the tentative diagnosis of HAE was ruled out and treatment with prednisolone 50 mg o.d. was initiated. He responded well to therapy and was discharged 9 days later with normal platelet count. The patient tapered prednisolone over 4 months and today, 1.5 years after the incidence, he has normal blood counts and is not specifically treated for ITP. He still receives Danazol 200 mg o.d. for HAE, which in fact may stabilize his ITP as well.

## 3. Discussion

In most cases, the attacks of HAE follow a predictable course. Many episodes are preceded by prodromal symptoms including a tingling or burning sensation in the affected area. In two-thirds of the patients, a nonpruritic serpiginous erythematous rash on the trunk, arms, or legs referred to as erythema marginatum may appear as part of the prodrome [10, 11]. Our patient did not experience any kind of prodromal symptoms other than a general discomfort. Swelling attacks in HAE manifest as recurrent local nonpitting, nonpruritic subcutaneous or submucosal edema [2, 12, 13]. Classically, the swelling develops gradually over a period of 12–24 hours and then slowly subsides within 72 hours. Severe attacks may last up to 5 days.

Any individual part of the integument can be affected but is most common in the extremities, abdomen, genitourinary system, and upper respiratory tract. Approximately 50% of the attacks involve the abdomen with severe abdominal pain, nausea, vomiting, and diarrhea as dominant symptoms [2]. Our patient complained about severe abdominal pain, but there was no pathology in routine laboratory test or at the abdominal CT. Episodes of swelling may also involve the upper respiratory tract, including the tongue, pharynx, and larynx. Our patient's main complaint was however swelling in the lower part of his face associated with difficulty in swallowing and development of a large haematoma of the jaw associated with multiple small petechiae on the extremities. These manifestations are not consistent with HAE and should lead the clinician to consider other differential diagnoses than angioedema swelling.

There are numerous inciting factors known to the attacks of HAE. Episodes may be triggered by minor trauma, surgery, dental treatment, psychological stress, or the use of certain medications. In many cases however, the attacks occur without any identifiable trigger [7].* Helicobacter pylori* infection is also considered among the causative factors [14].

The role of* Helicobacter pylori* is strongly proven and an association between chronic* Helicobacter pylori* infection and the occurrence of ITP has been found [15, 16]. Whether this Gram-negative bacterium plays a pathophysiological link with a key role in the pathogenesis could be speculated. We would recommend our patient to be tested in nearest future.

The classical complement pathway is an important driver in the pathogenesis of HAE. Increasing evidence suggests a contribution of complement activation in ITP [17, 18], the additional diagnosis for our patient. It could be considered that the disturbance of the coagulation system might increase the consumption of C1 and C1-INH and hereby act as a contributing cause in the development of HAE attacks. A correlation between ITP and angioedema has however not yet been described in the literature.

As illustrated in the disease history of our patient, onset of symptoms in HAE typically occurs in childhood and accelerates during adolescence. Despite the early onset in life, some patients are not diagnosed until adulthood, as there often is a significant diagnostic delay [3, 19].

The diagnosis of HAE should be suspected based on a history of recurrent attacks of angioedema or abdominal pain without associated urticaria [20]. Often the patient reports a family history of the condition but as 25% of the cases are caused by spontaneous mutations, having no family history does not rule out the diagnosis [13]. Laboratory testing is essential and required to confirm the diagnosis [4, 13]. It is not necessary to make an extensive paraclinical investigation every time the HAE patient is hospitalized, as most patients are self-administrating their attacks at home without any laboratory tests. Nevertheless, it is important to remain critical when something in the clinical demonstration does not agree with the overall picture. The diagnosis of ITP had already been demonstrated in the patient a decade earlier but it was not before the laboratory test had been performed that the diagnosis was reconsidered.

The therapeutic treatment of HAE can be divided into two regimens: the management of acute attacks and long-term prophylaxis [20, 21]. The treatment of choice in an acute attack consists of replacement with C1-INH concentrate (plasma-derived: Berinert, Cinryze or recombinant: Ruconest) and bradykinin B2 receptor antagonist (Firazyr) or, if those are unavailable, fresh-frozen plasma (contains C1-INH) [20–22]. Future attacks can be prevented by the use of attenuated androgens and the drug most frequently used is Danazol [21]. Although long-term prophylaxis with attenuated androgen is effective, it must be regarded critically due to a severe profile of side effects. Therapy with Danazol can be hepatotoxic and affect serum lipid levels. Hypertension, weight gain, acne, virilization, menstrual irregularities, and depression are also common [20, 21, 23]. More rare side effects as haematuria have been demonstrated [24]. Therefore, the microscopic haematuria found in this patient could be a result of the treatment although his low platelet count is more likely the cause. Due to the adverse event profile, all patients treated with Danazol must be monitored every six months with blood tests, urinalysis, and liver spleen ultrasound [23] as performed in our patient. Danazol is not only effective as long-term prophylaxis in HAE but also a good alternative therapeutic approach as treatment in ITP [25, 26]. This may explain why the patient did not have any symptoms of ITP for a long period of time as it turns out that he was possibly treated for both diseases. The side effects of Danazol are known to be dose dependent and therefore the dose had cautiously been reduced a few years earlier to achieve the lowest recommended effective dose at 200 mg daily [21]. When we tried a further dose reduction, he experienced the reported incident and had a relapse of ITP.

The diagnosis of HAE is often overlooked, as many of its symptoms mimic those of several other common conditions which is demonstrating diffuse swelling and abdominal discomfort [27, 28]. However, the clinical challenge is also seen the other way around. Swellings in HAE patients do not always have to be caused by angioedema but can relate to other concomitant disorders as demonstrated in this case. In fact, it is commonly seen that less experienced clinicians can have difficulties in looking beyond a rare initial diagnosis as they are concentrating too much on making the symptoms fit the original diagnosis. A thoroughly clinical examination in HAE patients is therefore essential like in other patients, giving the physicians the opportunity of looking outside the box and avoiding mental shortcuts [29]. If the clinical picture does not fit, it is most likely not the right diagnosis.
